# The Localisation of Sensation in the Brain

**Published:** 1894-05-19

**Authors:** 


### The Localisation of Sensation in the Brain.

There are few departments of medicine more interesting than the study of the functions of the cerebral cortex. As is well known, there is an area on either side of the fissure of Rolando, which presides over the motion of the opposite half of the body, and for a long time it was considered that this was the only function of that part, and some authorities, as Dr. Ferrier, think so still. This view was the usual view in England till quite recently, chiefly as a result of the experimental work of Ferrier, Horsley, Schäfer, and others, but abroad many authorities, and in England Dr. Bastain, always considered that this area had sensory as well as motor functions.

The exact variety of sensory attribute was differently stated by different observers; some thought that the muscle sense was represented in the motor area of the cortex, others believed that ordinary sensation was represented there, whilst others again were either vague in their statements, or gave no definite opinion. The school that considered that the area on either side of the fissure of Rolando is entirely and purely motor located all sensory impressions in other parts of the cortex, Ferrier thinking that the sensory area was in the hippocampal gyrus, and Horsley and Schäfer placing it in the gyrus fornicatus; but of these observers only Ferrier holds to his original statement. We thus see that experimentalists nearly all allow some sensory function to the motor area.

Turning now to the clinical side of the question, one of the chief workers is Dana, and he collected 142 cases of lesion of the motor area, and twenty cases of lesion of the gyrus fornicatus. He says: "I am certain no amount of scrutiny can explain away the numerous cases in which superficial cortical lesions have caused mono-plegias and mono-anæsthesias." He here refers to lesions near the motor area. But surgical cases are the most important, for in them a definite part of the brain is excised, while in patients who have a tumour, the surrounding inflammation, softening and pressure, have to be taken into account. Dr. Mott, speaking of surgical cases, says Horsley has had five cases in which large portions of the brain in the Rolandic area have been removed, and in all the operation has been followed by sensory defects. Starr, from an experience of more than thirty cases of cerebral operation, considers that there are centres for the reception of tactile sensation in the motor area of the cortex. Italian observers have recorded a case in which a tumour and a portion of the brain the size of a hen's egg were removed from the motor area. Tactile, general, thermal, and even painful sensations, which before the operation were intact, have been affected since the operation over the whole of the left half of the body except the face. It is true that some cases have been recorded in which the removal of part of the motor area of the cortex was not followed by any loss of sensation, but then it has always been found that the area removed has been small, or the tests have not been applied till some days after the operation.

From a consideration of the experimental and clinical evidence, therefore, we may conclude that the removal of a large portion of the motor area of the cortex leads to loss of sensation on the opposite side of the body, but that the removal of a small portion does not, and that the loss of sensation may soon disappear. We know that removal of even a small part from the motor cortex may lead to permanent paralysis. Thus we see that even if the same area of the cortex subserves both motor and sensory functions, yet there are considerable differences. These are probably to be explained, as Mott suggests, by the fact that the whole axis cylinder with its cell is removed in the case of the motor structures, but that only some of the terminal spreading branches of the sensory axis cylinders are removed. To make this clearer, we may compare both motor and sensory fibres to a tree, but the motor trunk springs in the cortex and the branches of it are peripheral, in the spinal cord, but in the case of the sensory fibres, the trunk comes upwards through the internal capsule, and only the branches are found in the cortex.

Now that we have learnt the position of the motor and sensory areas of the cortex, let us see if we can trace the paths to and from those areas. It is well known that motor impulses pass down from the motor area through the corona radiata, internal capsule, crus, pons, and medulla, and there decussating pass chiefly down the lateral tracts of the opposite side of the spinal cord. The terminal branches of these fibres transmit impressions to the nerve cells of the anterior cornua, from which they pass to the motor nerves and the muscles. Sensory impressions had better be traced upwards. They enter the cord by the posterior roots. Some fibres of this branch and the branches terminate in an arborization round a nerve cell soon after they enter the cord, others pass up in the columns of Goll and Burdach, and terminate in the nuclei of these columns in the medulla. The majority of these fibres do not decussate in the cord, but decussate in the sensory decussation in the medulla, which takes place after the nuclei of the columns of Goll and Burdach have been passed. After the decussation the fibres ultimately enter the fillet, and that passes up in the internal capsule at its posterior part, and so reaches the Rolandic area of the cortex. The impressions which pass up by this route are chiefly those of muscle-sense, and touch.

The most recent work on the subject is by Dr. Mott, and his experiments, which were very carefully performed, entirely confirm the view above given; for the excision of the motor area of seven monkeys invariably led to some loss of sensation in the paralysed part if animals who showed differences of sensation easily had been chosen. All the evidence, therefore, that can be collected goes to prove the truth of our statement that some forms of sensation are represented in the motor area of the cortex. This is a fact of the profoundest importance both to physicians, psychologists, and physiologists. Incidentally we have been able to point out that sensory function is less definitely localised than motor, and we have been able to describe the path downwards of motor impulses, and the path upwards of certain sensory impulses.

